# Trends in Co-Prescribing Opioids and Gabapentinoids Among Medicare Beneficiaries, 2017 to 2022

**DOI:** 10.3390/medsci14030345

**Published:** 2026-06-25

**Authors:** Mukaila Raji, Aashnika Sujit, Jordan Westra, Shilpa Rajagopal, Yong-Fang Kuo

**Affiliations:** 1 Division of Geriatrics, Department of Internal Medicine, University of Texas Medical Branch (UTMB), Galveston, TX 77555, USA; 2 John Sealy School of Medicine, University of Texas Medical Branch (UTMB), Galveston, TX 77555, USA; aasujit@utmb.edu (A.S.); shrajago@utmb.edu (S.R.); 3 Department of Epidemiology, School of Public and Population Health, University of Texas Medical Branch (UTMB), Galveston, TX 77555, USA; jrwestra@utmb.edu; 4 Department of Biostatistics & Data Science, School of Public and Population Health, University of Texas Medical Branch (UTMB), Galveston, TX 77555, USA; yokuo@utmb.edu

**Keywords:** co-prescribing, opioids, gabapentinoids, medicare, trends, older adults, prescriptions, geriatrics, long-term opioid use, chronic pain

## Abstract

Background: Co-prescribing opioids and gabapentinoids (GABA, gabapentin and pregabalin) is associated with increased risk of falls, fractures, opioid overdose and deaths. The Centers for Disease Control and Prevention (CDC) in 2016 and the Food and Drug Administration (FDA) in 2019 recommended caution in such co-prescribing. A key step in updating policy and revising prescribing guidelines aimed at reducing opioid and GABA co-use and its associated consequences is a thorough understanding of the prescriber and the patient factors associated with co-use. We thus examined national trends and patterns in opioid and GABA co-prescribing among Medicare beneficiaries from 2017 to 2022. Methods: We conducted a retrospective study of Medicare beneficiaries with ≥90 consecutive days of opioid use from 2017 to 2022. The study outcome was GABA use during the 90-day opioid use episode. A multivariable logistic regression model was constructed to examine the patient, prescriber and prescription factors associated with receiving a GABA prescription. Results: Our sample comprised 8035 opioid-only and 2818 opioid and GABA users. Non-cancer (e.g., back and neuropathic) pain was a more common diagnosis in the opioid and GABA cohorts than in the opioid-only cohorts. The opioid-GABA co-prescribing rate did not substantially change (2017: 24.5%, 2019: 28.2% and 2022: 25%). Co-prescribing rates were higher in non-white patients, those on Medicaid and Medicare, and those whose initial Medicare entitlement was not based on age. Tramadol and hydrocodone were the most prescribed opioids. Approximately 33% of opioid and GABA users started with an initial daily GABA dose of ≥1200 mg. In the 12-month lookback period, patients on opioids and GABA had nearly 17 clinic visits to approximately 8 different providers. Factors associated with co-prescribing were seeing pain physicians (odds ratio = 1.29, 95% confidence interval-[CI] = 1.11–1.50), having more healthcare encounters (6–11 visits, odds ratio-[OR] = 1.19, 95% CI = 1.02–1.39; 12–19, OR = 1.20, 95% CI = 1.00–1.43; 20+, OR = 1.27, 95% CI = 1.03–1.57) and seeing >10 providers (OR = 1.40, 95% CI = 1.12–1.73). Conclusions: One in four Medicare beneficiaries with long-term opioid use received opioid and GABA prescriptions. Our findings of association in co-prescribing with multiple visits to different clinics/prescribers can inform the development of public health policy and practice guidelines (e.g., prescription-drug monitoring program checks within electronic medical records, EMR alerts with opioid and GABA co-prescribing) to potentially reduce opioid and GABA prescriptions and associated adverse outcomes.

## 1. Introduction

Opioid-related morbidity and mortality is an increasing public health problem which has led to myriad government, insurance and health system changes to reduce opioid overprescribing [1,2,3]. Accompanying these changes is a shift by clinicians towards recommending non-opioid analgesic prescriptions as a presumably safer alternative to prescription opioids. This has led to a huge increase in prescribing gabapentinoids (GABA, gabapentin and pregabalin) in clinics, hospitals and post-acute care facilities [4,5,6,7]. Yet, instead of being opioid alternatives, GABA is increasingly being co-prescribed with opioids [5,6]. Opioid and GABA co-prescribing is associated with an increase in the risk of falls, fractures, respiratory depression, opioid overdose and deaths [8,9,10,11,12,13]. Using 2011–2016 fee-for-service Medicare data (n = 71,005 beneficiaries) for chronic pain, Zhou and colleagues (2021) found that the co-use of opioids and GABA was associated with twice the risk of drug overdose compared with opioid-only early discontinuers [10].

Because of the known toxicity of opioid and GABA co-use, both the Centers for Disease Control and Prevention (CDC) and the Food and Drug Administration (FDA) recommend caution in co-prescribing two such central nervous system (CNS) -depressant medications [3,14]. Unclear, however, is the extent to which opioid and GABA co-prescribing has changed since the 2016 CDC Guideline for Prescribing Opioids Policy and the 2019 FDA Drug Safety Communication about the dangers of the co-use of multiple CNS-depressant medications. A key step in updating policy and revising prescribing guidelines aimed at reducing opioid and GABA co-use and its associated consequences is a thorough understanding of the prescriber and patient factors associated with co-use. Gaps exist in the knowledge on the type, specialty and number of prescribers seen by patients; on the type, dose and duration of co-prescribed opioids and GABA; and on the patient behaviors and characteristics associated with opioid and GABA co-use. The current study addresses these gaps by using 2017 to 2022 national Medicare data to examine trends and patterns in prescribing opioid and GABA among long-term opioid users, particularly after the release of the CDC 2016 Opioid Policy Guideline and the 2019 FDA Drug Safety Communication. Because of the recent increase in the prescribing of GABA as an opioid alternative especially among long-term prescription opioid users, we decided to focus on the factors associated with prescribing GABA to long-term opioid users—a practice inconsistent with CDC and the FDA guidelines. Understanding the practice and policy-actionable drivers of the use of two or more CNS-depressing drugs can guide clinical practice guideline revision and inform public health policy aimed at reducing opioid-related morbidity and mortality.

## 2. Methods

### 2.1. Data Source

This retrospective cohort study used a 20% national sample of Medicare beneficiaries between 2017 and 2022. Medicare insurance covers hospital admission (Part A), provider visits and outpatient care (Part B) and prescription medications (Part D). Qualification is either by age (65 and older) via disability (<65). It does not pay for dental, vision or hearing services. The Medicare files used included the Master Beneficiary Summary Files (MBSF) and Claims files, including Medicare Provider and Analysis Review Files, Carrier Claims, Outpatient Standard Analytic Files and Part D Event Files (PDE) for all years. The MBSF file contained the demographic and enrollment information and the PDE contained the prescription information that was used to create the cohort. Medicare data were obtained from the United States of America (USA) Centers for Medicare and Medicaid Services and analyses were conducted at the Virtual Research Data Center. This study was approved on 23 July 2019 by the institutional review board (IRB # 16-0247) at the University of Texas Medical Branch.

### 2.2. Study Cohort

**Figure 1:**
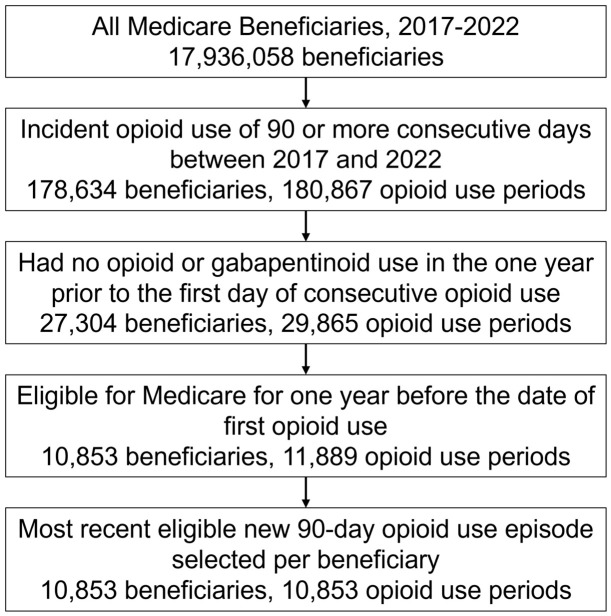
Study cohort selection.

The study included all Medicare beneficiaries who had at least one period of 90 consecutive days of opioid use between 2017 and 2022. The calculation of 90 days allowed for no gaps between the end of one prescription period and the beginning of another and did not extend prescriptions that were filled prior to the end of a previous prescription. This conservative approach to calculating 90 consecutive days ensured that all beneficiaries included in the cohort had at least 90 days of filled prescriptions. Excluded from this group were those who had any opioid or GABA prescription in the one year prior to the 90-day period, those who did not have complete Medicare enrollment (parts A, B and D, with no Medicare Advantage plan) in the year prior to the 90-day period and those who did not live in the 50 states or District of Columbia at the time of their initial opioid prescription. Due to the nature of the lookback (12 months), it was possible for a beneficiary to have multiple periods of 90+ days of consecutive opioid use that met all inclusion/exclusion criteria (e.g., one period in 2017 and another in 2020). In such cases, the most recent 90-day period was used. Figure 1 shows the process of determining the study cohort. The specific opioid types included for analysis in this study were tramadol, buprenorphine, hydrocodone, hydromorphone, morphine, oxycodone and codeine.

### 2.3. Study Measures

The measured outcome of this study was GABA use during the 90-day opioid use episode, and only prescriptions with a fill date that occurred during that period were counted. This was measured as any prescription filled during the 90-day period. Because many of the measures used in this study applied to both opioid and GABA prescriptions, beneficiaries in this study were classified as opioid-only or opioid and GABA.

Other variables of interest included those describing the patient, prescription and prescriber characteristics. Patient characteristics included age, sex, race, year of 90-day episode, original Medicare entitlement reason (old age or disabled/end-stage renal disease [ESRD]), Medicaid eligibility, pain-related diagnoses in the year prior to the 90-day period (abdominal/chest, arthritis, back, cancer, chronic, fractures, headaches, musculoskeletal, nerve, other, visceral and wound) and diagnosis of epilepsy or post-herpetic neuralgia. Associated diagnoses were determined using the International Classification of Diseases (ICD-10) codes. Prescription characteristics included opioid dosage (morphine milligrams equivalent [MME] per day), GABA dosage per day, prescription days of supply, total GABA days (for the opioid plus GABA group) and total number of prescriptions (overall and for each drug). Prescriber characteristics included prescriber specialty type (primary care, pain, nurse practitioner [NP], physician’s assistant [PA], other non-physician or other physician), number of prescribers during the 90-day period, number of specialties consulted during the 90-day period and number of prescriptions from each provider specialty type. Also included were characteristics from the 12 months prior to the 90-day period. These characteristics included the number of healthcare visits, number of providers seen, number of specialties seen, number of visits to the original prescriber (first opioid prescriber for the 90-day period), the most visited provider specialty type and whether the patient saw each provider specialty type.

### 2.4. Statistical Analysis

Descriptive statistics for all variables were presented to compare those who used opioids only and those who used opioids and GABA. These comparisons included all of the patient, prescription and prescriber characteristics described under the study measures. A Cochran–Armitage trend test was used to assess the change in GABA usage between 2017 and 2022. To test the association between the selected variables and the outcome of the receipt of a GABA prescription, a logistic regression model was used to calculate the odds ratios (ORs) for GABA use. Variables included in the model were age, sex, race, original Medicare entitlement reason, dual Medicaid eligibility, specialty of the first opioid prescriber, length of first opioid prescription, specialty of the most-seen provider in the lookback, seeing the original opioid prescriber in the lookback, individual indicators for each specialty seen in the lookback, number of visits in the lookback, number of providers seen in the lookback and number of visits in the 7 days before opioid initiation. All analyses were conducted using SAS Enterprise Guide 7.1 (SAS Inc., Cary, NC, USA).

## 3. Results

During the study period, the opioid and GABA co-prescribing rate remained similar across the years: 24.5% in 2017, 27.02% in 2018, 28.2% in 2019, 25.9% in 2020, 25.5% in 2021 and 25% in 2022 (Cochran–Armitage trend test *p* = 0.9225). The demographics of patients receiving opioids only versus opioid and GABA prescriptions were relatively comparable by age, race and sex, with a majority of patients identifying as white and female (Table 1). Key pain diagnoses in the year prior to opioid use more common among those with opioid-only prescription utilization were back pain (55.6%), arthritis (40.6%) and chronic pain (37.2%). These primary diagnoses differed among patients co-prescribed opioid and GABA, with 69.9% of this group having back pain, 51.6% having chronic pain and 44.6% having arthritis. Of note, the co-use of opioid and GABA was more common in those with diagnoses of back pain, chronic pain and nerve pain, while opioid-only use was more common among persons with diagnoses of cancer and visceral pain.

For both the opioid-only and opioid and GABA cohorts, the initial opioid prescription comprised a low daily MME, with over 80% in each group receiving a first opioid prescription of <50 MME/day (Table 2). When comparing this to the MME/day average across all opioid prescriptions for the two cohorts, the percentage of prescriptions of <50 MME/day continued to remain the predominant dose type (71.82% of opioid-only prescriptions and 66.9% of opioid + GABA prescriptions). Strikingly, daily dosing of the initial GABA prescription in the opioid and GABA cohort was more variable, with 36.3% of these first-time prescriptions containing a daily GABA dose of <600 mg, 30.5% containing a daily GABA dose of 600–1199 mg and 33.1% containing a daily GABA dose of ≥1200 mg.

Across a 90-day opioid period, a 30-day supply period was the most frequent duration given for an initial prescription for both the opioid-only (41.1%) and opioid and GABA (47.4%) medications (Table 3). Of note, nearly 25% of first-time opioid-only prescriptions were allocated for >30 days, referred to as “long opioid prescriptions,” compared to approximately 15% of initial opioid and GABA prescriptions. In this group of long opioid prescriptions, the median prescription supply length was 90 days, and tramadol hydrochloride constituted the majority (65.7%) of these prescriptions (Supplemental Table S1). Among those with opioid and GABA prescriptions, 62.8% of the first GABA prescriptions were for 30 days, 24.5% were >30 days and 12.7% were for <30 days.

In general, primary care physicians were the most common prescriber of opioid-only (45.4%) and opioid and GABA (43.9%) prescriptions, followed by NPs (18.1% for opioid-only and 16.7% for opioid plus GABA) (Table 3). Compared to other provider groups, primary care physicians also represented a larger number of first prescribers of opioid-only and opioid and GABA prescriptions. While primary care physicians prescribed the most initial and refill prescriptions of opioid-only and opioid and GABA medications, slight increases were seen in the percentages of NPs prescribing refills across both cohorts (Supplemental Table S2).

Additionally, differences were observed in the number of prescribers for opioid-only versus opioid and GABA prescriptions in a 90-day period. Across the cohort of patients receiving opioids only, 70.5% had a single provider and 20.7% had two providers prescribing their opioid medications in a 90-day period (Table 3). Approximately 5.6% of these patients received their opioid prescriptions from three providers over 90 days. Comparatively, approximately 40.1% of patients receiving opioids and GABA had one prescriber, while 35.2% had two prescribers and 15.4% had three providers within a 90-day period. When specifically examining the opioid and GABA cohort, 52.6% received their GABA prescription from the same prescriber of their original opioid prescription. Moreover, 4.6% of patients using opioids and GABA received their GABA prescription on the same day as their original opioid prescription, the majority of which came from a primary care physician (Table 3, Supplemental Table S3). The most common prescriber combinations for the first opioid and GABA prescriptions were primary care physicians and pain physicians (Supplemental Table S4).

Over a 12-month lookback period of general visits prior to their first opioid prescription, patients who were co-prescribed opioids and GABA had, on average, nearly seventeen visits, seeing approximately eight different providers (Table 4). On the other hand, patients receiving only opioids had slightly fewer visits (fourteen) and provider types (nearly seven) during this period. Among these average numbers of visits, approximately three were to the patient’s original prescriber of opioid or opioid and GABA identified within the 90-day period. Moreover, an estimated 35.3% of patients prescribed opioids only and 37.7% of patients co-prescribed opioids and GABA did not see their original opioid prescriber in the 12 months prior to receiving their first opioid prescription. For these patients, their original opioid prescriber was most commonly a primary care physician (Supplemental Table S5).

The initial prescriber has an impact on GABA prescriptions in a cohort of long-term (at least 90 days) opioid users (Table 5). In comparison to primary care physicians as the first prescriber, patients seeing pain specialists (OR 1.37, 95% confidence interval [CI] = 1.16–1.62) had higher odds of receiving GABA prescriptions. In the 12-month lookback period, patients also had higher odds of receiving GABA prescriptions if they saw a pain physician (OR 1.29, 95% CI = 1.11–1.50), had more health encounters (6–11 encounters, OR 1.19, 95% CI = 1.02–1.39; 12–19 encounters, OR 1.20, 95% CI = 1.00–1.43; and 20+ encounters, OR 1.27, 95% CI = 1.03–1.57) or saw more than 10 providers (OR 1.40, 95% CI = 1.12–1.73).

## 4. Discussion

Our findings can be summarized as follows: the number of patients with any opioid prescription (alone or in combination with GABA) declined from 2017 to 2022, starting soon after the 2016 CDC Guideline for Prescribing Opioids for Chronic Pain and the 2019 FDA drug safety warnings [3,14]. Tramadol was the most prescribed opioid among long-term opioid users, consistent with other research findings showing increasing trends in tramadol prescribing (in part due to various opioid-restricting policies) and a concomitant rise in tramadol-related toxicities (e.g., death, hypoglycemia and hospitalization) [15,16,17].

However, the opioid and GABA co-prescribing rate remained essentially unchanged from 2017 to 2022 (24.5% in 2017 and 25% in 2022), despite the 2016 CDC opioid prescribing guideline and the 2019 FDA drug safety warnings [3,14]. Co-prescribing rates were higher in non-white patients, those on Medicaid and Medicare (dually eligible) and those whose initial Medicare entitlement was based on disability or ESRD, a population at high risk of opioid overdose death [18]. One in four Medicare beneficiaries on long-term opioids received GABA, despite the FDA/CDC warnings and the increasing evidence the high risk of opioid-related overdose, falls and fractures in patients on both drugs [3,11,14,19]. This finding underscores the need for a public health policy update and clinical guideline reform.

Opioid-only users had higher rates of cancer diagnoses than opioid and GABA users. The opioid and GABA users, however, had higher rates of back pain, chronic pain and nerve pain diagnoses. The high opioid and GABA co-prescribing rate for non-cancer pain underscores the need for multidisciplinary (pain, mental health and primary care providers) and multimodal approaches to pain management, including the use of pharmacological (opioids and non-opioids), non-pharmacological (e.g., physical therapy and nerve stimulation) and local injections [20].

Consistent with the CDC guidelines for a low opioid starting dose, over 80% of both cohorts started with a low daily MME dose (<50 MME/day) as their initial opioid prescription. On the other hand, approximately 33% of the opioid and GABA user cohort started with a high initial daily GABA dose of 1200 mg or higher, a dose associated with falls/fractures, delirium and hospitalization in older adults [21,22]. Although prevalence was low at 4.0%, prescribing GABA and opioids on the same day of a visit carries a particularly high risk of drug overdose and deaths, and such practice should be avoided [11,19].

Primary care physicians were the most common prescribers of opioid and GABA (43.9%), followed by NPs (16.7%), with modest increases over time in the percentage of NPs and PAs prescribing opioid and GABA refills. In the 12-month lookback period, patients on opioids and GABA had nearly seventeen clinic visits to approximately eight different providers, with approximately three of the visits to the patient’s original prescriber of opioid or opioid and GABA, thus increasing the fragmentation and discontinuity of care which serve as major contributors to polypharmacy, medical errors, potentially inappropriate medication, misdiagnosis and death [23,24]. Approximately 20.5% of the opioid-GABA co-users visited four different specialties in twelve months. Such multiple visits to different sites, providers and specialties may have been due to myriad reasons, including but not limited to healthcare system fragmentation with frequent changes in insurance or in providers, challenges in accessing optimal pain treatments leading to undertreated pain, socioeconomic barriers to accessing continuity of care, complexity of pain management needs and a lack of coordination among providers. Given these circumstances and the need to reduce the rates of GABA-opioid co-prescribing (and the associated adverse outcomes), future research should examine avenues to effectively identify controlled substance utilization, such as routine use of prescription drug monitoring program (PDMP) checks by prescribers. These checks may show the number of prescribers from whom a patient has received GABA-opioids as well as other controlled substances, helping obtain a more comprehensive picture of a patient’s prescription history and risk status [3,25]. One approach to facilitate and encourage PDMP checking by clinicians is by integrating the PDMP into the electronic health record (EHR), though additional research is needed to identify more streamlined methods for accessing PDMP data across different states and health systems to promote alignment in care outcomes [26].

## 5. Limitations

This study had limitations. First, we used data from Fee-for-Service Medicare beneficiaries, and our results may not be generalizable to Medicare Advantage or Health Maintenance Organization populations. The calculation of a 90-day consecutive period of opioid use without gaps between prescription periods as defined in the study criteria may also not have fully accounted for patients who experienced delays in prescription re-fills. Additionally, we could not ascertain whether the patients actually took the prescribed opioids and/or GABA, as we analyzed only PDE files. We also had no data on non-prescribed drugs (e.g., drugs received from friends, the street or mail orders) that patients might have obtained outside of Medicare. A limitation of using claims data is the inability to confirm whether patients with opioid medications are actually using them or not, such that starting a new GABA medication prescription during the last month of an opioid prescription might actually be a switch instead of concurrent use. This limitation could have led to the possible misclassification of “less than 30 days” of GABA/OPIOID overlap as concurrency when it could have been switches. Because Medicare claims data are constrained in addressing this misclassification, one way to address this is a qualitative study approach or a patient survey to confirm actual use, discontinuation or concurrency. Our study did not address dynamic changes in sites, sources and severity of pain over time—an important consideration in explaining why additional GABA is prescribed, and this is an area for future study. The NP prescribing data might have been incomplete, as physicians may have included some NP effort as incidental billing, and this may likely have under-reported NP-specific data in the analysis. While our study focused on factors associated with the prescribing of GABA to long-term opioid users—a practice inconsistent with CDC and the FDA guidelines—future studies are needed to also examine the reverse: new opioid prescribing for long-term GABA users. Another area for future study is an examination of whether the decision by clinicians to add or not add GABA to long-term opioid (LTOP) users is influenced by whether the LTOP user is on other CNS-active medications such as benzodiazepine and Z drugs.

## 6. Conclusions

The co-prescribing of opioids and GABA in the Medicare population—a risk factor for falls, fractures and opioid overdose—is a common and potentially modifiable public health problem. One in four Medicare beneficiaries on long-term opioids were also on GABA medications. The predictors of being on combined opioid and GABA prescriptions include having multiple visits to different clinics, providers and specialties; having both Medicare and Medicaid (dual eligibility); and having qualified for Medicare via disability or ESRD. These findings suggest the need for further research assessing the efficacy of clinical practice interventions (e.g., integrating the PDMP within electronic health record (EHR) systems and incorporating EHR alerts when opioid and GABA are co-prescribed) in helping to reduce co-prescribing two CNS depressant drugs (opioid and GABA) among Medicare patients and decreasing associated morbidity. Designing and implementing targeted, patient-centered educational materials focused on the risks of opioid-GABA co-use for Medicare patients and caregivers have the potential to engage patients and their care partners in the optimal and safe management of their painful conditions.

## Figures and Tables

**Table 1:** Beneficiary characteristics by opioid/GABA use group.

		Opioid Only		Opioid + GABA		
		N	%	N	%	*p*-Value
Total		8035	100%	2818	100%	
Age	Mean, SD	70.42	14.45	67.03	14.75	
	<65	2218	27.6%	1078	38.3%	<0.0001
	65–69	1305	16.2%	431	15.3%	
	70–74	1283	16.0%	426	15.1%	
	75–79	1025	12.8%	316	11.2%	
	80–84	845	10.5%	234	8.3%	
	85+	1359	16.9%	333	11.8%	
Sex	Male	3338	41.5%	1180	41.9%	0.7595
	Female	4697	58.5%	1638	58.1%	
Race	White	6660	82.9%	2293	81.4%	0.0752
	Black	661	8.2%	248	8.8%	
	Other	210	2.6%	77	2.7%	
	Asian	114	1.4%	54	1.9%	
	Hispanic	390	4.9%	146	5.2%	
Original Entitlement	Old Age	4622	57.5%	1348	47.8%	<0.0001
	Disabled/ESRD	3413	42.5%	1470	52.2%	
Dual Medicaid Eligible	No	4633	57.6%	1373	48.7%	<0.0001
	Yes	3402	42.4%	1445	51.3%	
Pain Locations	Abdominal/Chest Pain	2748	34.2%	1101	39.1%	<0.0001
	Arthritis	3263	40.6%	1256	44.6%	0.0002
	Back Pain	4464	55.6%	1971	69.9%	<0.0001
	Cancer	1509	18.8%	470	16.7%	0.0129
	Chronic Pain	2989	37.2%	1455	51.6%	<0.0001
	Fractures	1148	14.3%	413	14.7%	0.6317
	Headaches	1365	17.0%	606	21.5%	<0.0001
	Musculoskeletal	1696	21.1%	746	26.5%	<0.0001
	Nerve Pain	1278	15.9%	918	32.6%	<0.0001
	Other Pain	1595	19.9%	591	21.0%	0.2014
	Visceral Pain	832	10.4%	279	9.9%	0.4939
	Wound Pain	643	8.0%	232	8.2%	0.6992
Other Diagnoses	Epilepsy	312	3.9%	108	3.8%	0.9048
	Post-herpetic neuralgia	6	0.1%	1	0.0%	0.4808

GABA: gabapentinoids; SD: standard deviation; ESRD: end-stage renal disease.

**Table 2:** Opioid and GABA dosage information, by opioid/GABA use group.

		Opioid Only		Opioid + GABA	
		N	%	N	%
First Opioid Prescription MME/day	<50	6528	81.2%	2269	80.5%
	50-<90	414	5.2%	174	6.2%
	90+	1093	13.6%	375	13.3%
All Opioid Prescriptions MME/Day—average across all prescriptions	<50	5770	71.8%	1884	66.9%
	50-<90	829	10.3%	372	13.2%
	90+	1436	17.9%	562	19.9%
First GABA Prescription * Dose/Day	<600			1024	36.3%
	600-<1200			860	30.5%
	1200+			934	33.1%
All GABA Prescriptions * Dose/Day—average across all prescriptions	<600			983	34.9%
	600-<1200			837	29.7%
	1200+			990	35.1%

GABA: gabapentinoids; MME: morphine milligrams equivalent. * GABA dose is in milligram (mg).

**Table 3:** Prescription and prescriber characteristics in the 90-day long-term opioid period, by opioid/GABA use group.

		Opioid Only		Opioid + GABA	
		N	%	N	%
Total		8035	100%	2818	100%
First Prescription Prescriber Type	Unknown	59	0.7%	23	0.8%
	Primary Care	4098	51.0%	1245	44.2%
	Pain	718	8.9%	399	14.2%
	NP	1185	14.7%	398	14.1%
	PA	470	5.8%	200	7.1%
	Other Non-Physician	10	0.1%	3	0.1%
	Other Physician	1495	18.6%	550	19.5%
First Prescription Supply Days	<7	1339	16.7%	556	19.7%
	8–29	1405	17.5%	502	17.8%
	30	3301	41.1%	1335	47.4%
	31+	1990	24.8%	425	15.1%
All Prescriptions Prescriber Type	Unknown	313	0.9%	152	0.7%
	Primary Care	15,728	45.4%	9144	43.9%
	Pain	3342	9.6%	2438	11.7%
	NP	6279	18.1%	3477	16.7%
	PA	2201	6.4%	1547	7.4%
	Other Non-Physician	50	0.1%	42	0.2%
	Other Physician	6733	19.4%	4013	19.3%
All Prescriptions Supply Days	<7	4795	13.8%	2262	10.9%
	8–29	8512	24.6%	4430	21.3%
	30	18,360	53.0%	12,437	59.8%
	31+	2979	8.6%	1684	8.1%
Number of Prescribers in 90-Day Period	1	5665	70.5%	1130	40.1%
	2	1662	20.7%	991	35.2%
	3	451	5.6%	435	15.4%
	4	160	2.0%	167	5.9%
	5	61	0.8%	66	2.3%
	6	25	0.3%	13	0.5%
	7	9	0.1%	9	0.3%
	8	0	0.0%	5	0.2%
	9	1	0.0%	2	0.1%
	10	1	0.0%	0	0.0%
Number of Specialties in 90-Day Period	Unknown	40	0.5%	4	0.1%
	1	6288	78.3%	1460	51.8%
	2	1460	18.2%	1074	38.1%
	3	223	2.8%	247	8.8%
	4	21	0.3%	32	1.1%
	5	3	0.0%	1	0.0%
GABA Prescription from the Same Prescriber as Original Opioid Prescription		-	-	1482	52.6%
GABA Prescription from the Same Prescriber Specialty as Original Opioid Prescription		-	-	1795	63.7%
GABA Prescription on the Same Day as Original Opioid Prescription		-	-	129	4.6%
Number of Prescriptions	Mean, SD	4.29	2.85	7.34	3.56
RX by NP	Mean, SD	0.78	1.94	1.22	2.61
RX by Primary Care	Mean, SD	1.95	2.52	3.22	3.82
RX by PA	Mean, SD	0.27	1.11	0.54	1.78
RX by Pain	Mean, SD	0.41	1.32	0.86	2.07
RX by Other Non-Physician	Mean, SD	0.01	0.16	0.01	0.26
RX by Other Physician	Mean, SD	0.83	2.02	1.42	2.64
<7 Day Prescriptions	Mean, SD	0.59	1.87	0.79	2.22
8–29 Day Prescriptions	Mean, SD	1.05	2.02	1.56	2.71
30 Day Prescriptions	Mean, SD	2.28	2.04	4.39	2.83
31+ Day Prescriptions	Mean, SD	0.37	0.6	0.59	0.93
Number of Opioid Prescriptions	Mean, SD	4.29	2.85	4.83	2.81
Number of GABA Prescriptions	Mean, SD	0	0	2.51	1.63
GABA Days	Mean, SD	0	0	59.71	24.73

GABA: gabapentinoids; NP: nurse practitioner; PA: physician assistant; RX: prescription.

**Table 4:** Provider visit information for the 12-month lookback period, by opioid/GABA use group.

		Opioid Only		Opioid + GABA	
		N	%	N	%
Total		8035	100%	2818	100%
Number of Visits 12 Prior Months	Mean, SD	14.19	14.31	16.54	16.55
Had 0 visits in 12 Prior Months		373	4.6%	97	3.4%
Number of Providers 12 Prior Months	Mean, SD	6.85	6.92	8.07	7.82
Number of Visits to Original Prescriber (of 90-day period)	Mean, SD	3.09	4.71	2.97	4.83
Number of Visits to Most Visited Provider	Mean, SD	5.64	5.92	6.08	6.01
Number of Specialties 12 Prior Months	0	373	4.6%	97	3.4%
	1	1085	13.5%	339	12.0%
	2	2605	32.4%	798	28.3%
	3	2442	30.4%	869	30.8%
	4	1296	16.1%	577	20.5%
	5	229	2.9%	136	4.8%
	6	5	0.1%	2	0.1%
Most Visited Provider Type	Unknown	373	4.6%	97	3.4%
	Primary Care	3342	41.6%	1127	40.0%
	Pain	311	3.9%	129	4.6%
	NP	1043	13.0%	410	14.5%
	PA	370	4.6%	154	5.5%
	Other Non-Physician	20	0.2%	10	0.4%
	Other Physician	2576	32.1%	891	31.6%
Any Visit to Provider Type in Prior 12 Months	Primary Care	6197	77.1%	2225	79.0%
	Pain	1012	12.6%	522	18.5%
	NP	3611	44.9%	1404	49.8%
	PA	2416	30.1%	986	35.0%
	Other Non-Physician	101	1.3%	43	1.5%
	Other Physician	7016	87.3%	2459	87.3%
Had At Least 1 Visit to Original Prescriber (of the 90-day period)	Yes	5196	64.7%	1757	62.3%
	No	2839	35.3%	1061	37.7%

GABA: gabapentinoids; NP: nurse practitioner; PA: physician assistant; SD: standard deviation.

**Table 5:** Multivariable analysis of the odds ratios of gabapentinoid use among long-term opioid users.

Variable	Level	Adjusted OR (95% CI)
Age	<65	Ref
	65–69	0.74 (0.63, 0.87)
	70–74	0.77 (0.64, 0.91)
	75–79	0.69 (0.57, 0.83)
	80–84	0.61 (0.49, 0.75)
	85+	0.53 (0.43, 0.64)
Sex	Female	Ref
	Male	0.93 (0.85, 1.02)
Race	White	Ref
	Black	0.92 (0.78, 1.08)
	Asian	1.49 (1.06, 2.09)
	Hispanic	1.02 (0.83, 1.25)
	Other	0.96 (0.74, 1.26)
Original Medicare Entitlement	Disabled/ESRD	Ref
	Old Age	1.06 (0.92, 1.22)
Dual Medicaid Eligibility	No	Ref
	Yes	1.21 (1.09, 1.34)
First Prescriber	Primary Care	Ref
	Unknown	1.04 (0.63, 1.71)
	Pain	1.37 (1.16, 1.62)
	NP	0.92 (0.79, 1.06)
	PA	1.16 (0.96, 1.41)
	Other Non-Physician	0.73 (0.19, 2.77)
	Other Physician	1.10 (0.97, 1.24)
First Prescription Days	<7	Ref
	8–29	0.86 (0.75, 1.00)
	30	0.99 (0.87, 1.11)
	31+	0.66 (0.57, 0.77)
Most Seen Provider in Prior 12 Months	Primary Care	Ref
	Unknown	0.89 (0.67, 1.18)
	Pain	0.72 (0.56, 0.94)
	NP	1.10 (0.94, 1.29)
	PA	1.11 (0.88, 1.39)
	Other Non-Physician	1.36 (0.56, 3.33)
	Other Physician	1.01 (0.90, 1.14)
Saw Original Prescriber in the 12 Months Prior	No	Ref
	Yes	0.94 (0.85, 1.04)
Specialties Seen in Prior 12 Months (Yes/No)	Primary Care	1.06 (0.92, 1.22)
	Pain	1.29 (1.11, 1.50)
	NP	0.99 (0.88, 1.10)
	PA	1.03 (0.92, 1.14)
	Other Non-Physician	0.92 (0.61, 1.40)
	Other Physician	0.85 (0.73, 1.00)
Number of Visits in Prior 12 Months	≤5	Ref
	6–11	1.19 (1.02, 1.39)
	12–19	1.20 (1.00, 1.43)
	20+	1.27 (1.03, 1.57)
Number of Providers Seen in Prior 12 Months	≤3	Ref
	4–5	1.05 (0.89, 1.23)
	6–9	1.07 (0.90, 1.28)
	10+	1.40 (1.12, 1.73)
Number of Visits in Prior 7 Days	0	Ref
	1	0.98 (0.88, 1.09)
	2+	0.98 (0.84, 1.15)

GABA: gabapentinoids; NP: nurse practitioner; PA: physician assistant; OR: odds ratio; CI: confidence interval.

## Data Availability

The data used in this study are not publicly available and require a Data Use Agreement (DUA) from the Centers for Medicare & Medicaid Services.

## Supplementary Materials

The following supporting information can be downloaded at: https://www.mdpi.com/article/10.3390/medsci14030345/s1, Table S1: Long Opioid Prescriptions (31+ Days), Table S2: First Rx vs Refill Rx, Table S3: Prescriber Type of those who had Opioid and GABA on the same day (%), Table S4: First Opioid Prescriber and First GABA Prescriber, Table S5: Characteristics of those who did not see their original prescriber in the 12 months prior to the first opioid prescription.
