# Supplementary material for: Trends in Co-Prescribing Opioids and Gabapentinoids Among Medicare Beneficiaries, 2017 to 2022
Source: Med Sci (Basel). 2026 Jun 25;14(3):345. doi: 10.3390/medsci14030345 (PMC13413538; doi:10.3390/medsci14030345)
Supplement: Supplementary file 1 [file medsci-14-00345-s001.zip › medsci-4362689-supplementary.pdf]

Supplemental Table S1. Long Opioid Prescriptions (31+ Days)

Number of Days		
Median	90	
Q1	84	
Q3	90	
Max	473	
Prescriber Type		
Unknown	99	0.7%
Primary Care	9,033	63.9%
Pain	502	3.6%
NP	1,132	8.0%
PA	629	4.4%
Non	7	0.0%
Other	2,736	19.4%
Opioid Type		
Tramadol HCL	9,295	65.7%
Buprenorphine	262	1.9%
Hydrocodone	1,808	12.8%
Hydromorphone	54	0.4%
Morphine	297	2.1%
Oxycodone	980	6.9%
Codeine	531	3.8%

Supplemental Table S2. First Rx vs Refill Rx		Opioid Only				Opioid + GABA							
		Opioid Rx				Opioid Rx				GABA Rx			
		First		Refills		First		Refills		First		Refills	
Prescriber Type	Unknown	59	0.7%	254	1.0%	23	0.8%	103	0.9%	14	0.5%	12	0.3%
	Primary	4,102	51.1%	11,626	43.7%	1,242	44.1%	4,366	40.1%	1,421	50.4%	2,115	49.3%
	Pain	718	8.9%	2,624	9.9%	398	14.1%	1,453	13.3%	233	8.3%	354	8.3%
	NP	1,182	14.7%	5,097	19.2%	401	14.2%	1,933	17.8%	416	14.8%	727	17.0%
	PA	470	5.8%	1,731	6.5%	201	7.1%	858	7.9%	191	6.8%	297	6.9%
	Non	10	0.1%	40	0.2%	3	0.1%	17	0.2%	8	0.3%	14	0.3%
	Other	1,494	18.6%	5,239	19.7%	550	19.5%	2,160	19.8%	535	19.0%	768	17.9%
Days of Supply	<7	1,339	16.7%	3,456	13.0%	562	19.9%	1,391	12.8%	58	2.1%	251	5.9%
	8-29	1,410	17.5%	7,102	26.7%	495	17.6%	2,940	27.0%	300	10.6%	695	16.2%
	30	3,296	41.0%	15,064	56.6%	1,339	47.5%	6,289	57.8%	1,770	62.8%	3,039	70.9%
	31+	1,990	24.8%	989	3.7%	422	15.0%	270	2.5%	690	24.5%	302	7.0%

Supplemental Table S3. Prescriber Type of those who had Opioid and GABA on the same day (%)

Prescribed both on same day from same prescriber		
Unknown		1
Primary Care		60
Pain		7
NP		10
PA		7
Non		0
Other		29
Prescribed both on same day from different prescribers		
Opioid Prescriber	GABA Prescriber	
Unknown	Primary	1
Primary	Primary	2
Primary	Other	1
Pain	Primary	1
NP	Primary	1
NP	NP	1
PA	Primary	2
PA	NP	1
Other	Primary	4
Other	PA	1

Supplemental Table S4. First Opioid Prescriber and First GABA Prescriber

	GABA Prescriber						
Opioid Prescriber	Unknown	Primary Care	Pain	NP	PA	Non	Other
Unknown	8	8	1	1	2	0	3
Primary Care	2	991	25	73	44	3	107
Pain	1	118	175	46	16	1	42
NP	1	109	5	232	9	1	41
PA	0	50	7	19	90	0	34
Non	0	1	0	2	0	0	0
Other	6	136	16	57	25	3	307

Supplemental Table S5. Characteristics of those who did not see their original prescriber in the 12 months prior to the first opioid prescription.

		Opioid Only		Opioid+GABA	
		2,839	100%	1,061	100%
Original opioid prescriber specialty	Unknown	49	1.7%	21	2.0%
	Primary	1,140	40.2%	386	36.4%
	Pain	288	10.1%	175	16.5%
	NP	526	18.5%	160	15.1%
	PA	264	9.3%	99	9.3%
	Other Non-Physician	3	0.1%	2	0.2%
	Other Physician	569	20.0%	218	20.5%
Number of Visits 12 Prior Months	Mean, SD	10.01	12.55	12.2	14.93
Had 0 visits in 12 Prior Months		363	12.8%	95	9.0%
Number of Providers 12 Prior Months	Mean, SD	5.53	6.32	6.7	7.2
Number of Visits to Most Visited Provider	Mean, SD	3.9	5.54	4.37	4.68
Number of Specialties 12 Prior Months	0	363	12.8%	95	9.0%
	1	569	20.0%	188	17.7%
	2	813	28.6%	286	27.0%
	3	676	23.8%	292	27.5%
	4	364	12.8%	172	16.2%
	5	54	1.9%	28	2.6%
Most Visited Provider Type	Unknown	363	12.8%	95	9.0%
	Primary	787	27.7%	340	32.0%
	Pain	72	2.5%	30	2.8%
	NP	364	12.8%	141	13.3%
	PA	151	5.3%	60	5.7%
	Other Non-Physician	10	0.4%	3	0.3%
	Other Physician	1,092	38.5%	395	37.2%
Any visit to provider type in prior 12 months	Primary	1,617	57.0%	680	64.1%
	Pain	245	8.6%	124	11.7%
	NP	1,109	39.1%	482	45.4%
	PA	797	28.1%	329	31.0%
	Other Non-Physician	27	1.0%	14	1.3%
	Other Physician	2,517	88.7%	930	87.7%
